# Exploring the Challenges in Palliative Care As Perceived by the Saudi Physicians: A Cross-Sectional Study

**DOI:** 10.7759/cureus.66579

**Published:** 2024-08-10

**Authors:** Norah A Alhatim, Maied Z AlShehery

**Affiliations:** 1 Department of Palliative medicine, King Fahad Medical City, Riyadh, SAU; 2 Palliative Care Department, King Fahad Medical City, Riyadh, SAU

**Keywords:** multidisciplinary care, ethical dilemmas, telemedicine, covid-19, healthcare, saudi arabia, physicians, challenges, palliative care

## Abstract

Background: Palliative care is essential for managing patients with life-limiting illnesses. In Saudi Arabia, providing effective palliative care is uniquely challenging due to cultural, religious, and social factors. Despite healthcare advancements, there is a gap in understanding the challenges faced by physicians in palliative care within this context.

Aims: This study aimed to explore the challenges encountered in palliative care as perceived by physicians in Riyadh, Saudi Arabia.

Methods: A cross-sectional survey was conducted using an electronic questionnaire distributed among physicians involved in palliative care at the specified healthcare institutions. The questionnaire assessed their perceptions of the challenges in palliative care and the influence of their socio-demographic backgrounds on these perceptions. Data were analyzed using the Statistical Package for the Social Sciences (SPSS).

Results: The age distribution of the enrolled physicians showed that a majority were between 20-40 years old (48.48%, n = 96). Male physicians accounted for 64.65% (n = 128), and females for 35.35% (n = 70). Various specialties were represented, with critical care (15.66%, n = 31) and radiation oncology (16.67%, n = 33) being the most common. Major challenges identified included limited outpatient and inpatient services (54.55%, n = 108), restricted access to allied healthcare professionals (60.61%, n = 120), ethical dilemmas due to triaging (63.13%, n = 125), lack of telemedicine facilities (57.07%, n = 113), and the impact of the COVID-19 pandemic on pain and palliative care research (60.1%, n = 119). Strategies adopted to mitigate these challenges included creating triage systems (54.55%, n = 108), using telemedicine (60.61%, n = 120), advanced care planning (63.13%, n = 125), and providing necessary personal protective equipment (PPE) (60.1%, n = 119).

Conclusion: This study highlights significant barriers in palliative care, such as limited services, ethical dilemmas, and lack of telemedicine facilities. Addressing these challenges requires ethical support for healthcare providers, integration of telemedicine, continuous education, and improved access to multidisciplinary care teams, which are crucial for enhancing palliative care quality and ensuring comprehensive patient support.

## Introduction

Palliative care represents a paradigm of medical care focused on alleviating suffering and improving the quality of life for patients with serious illnesses [1]. This approach is holistic, addressing not only physical symptoms but also catering to psychological, social, and spiritual needs [2]. It is crucial to understand that palliative care is applicable at various stages of illness, often provided in conjunction with curative treatments, and is not limited to end-of-life care [3]. This introduction to palliative care sets the foundation for a comprehensive understanding of its role in the healthcare system [4].

The relevance of palliative care in today’s healthcare landscape is increasingly significant [5]. With a global rise in chronic illnesses and an aging population, the demand for palliative care services is intensifying [6]. This form of care is recognized as a fundamental patient right, essential for improving the quality of life for patients with life-limiting conditions [7]. The escalating need for palliative care underscores its importance in the broader context of global health [8].

In the realm of palliative care, physicians play a pivotal role [9]. They are not only caregivers but also experts in symptom management and empathetic communication, often coordinating with a multidisciplinary team [10]. This role requires a balance of medical expertise and patient-centered care, highlighting the unique position of physicians in palliative care [1]. Physicians are at the forefront of palliative care delivery, often making critical decisions that directly impact patient outcomes. Their extensive training and experience place them in a unique position to address the complex needs of palliative care patients. Furthermore, physicians are frequently the primary point of contact for patients and families navigating serious illnesses, making their ability to manage symptoms and provide compassionate care essential.

However, physicians in palliative care encounter a unique set of challenges, which form the core focus of this research. These challenges are diverse, encompassing emotional, clinical, ethical, and communicative dimensions. Specifically, this study investigates challenges at different levels, including hospital, personal, and home and hospice settings [2]. At the hospital level, challenges include limited outpatient and inpatient services, restricted access to allied healthcare professionals, ethical dilemmas due to triaging, lack of pharmacy dispensing wings, insufficient telemedicine facilities, restricted caregiver entry, and the impact of the COVID-19 pandemic on pain and palliative care research. At the personal level, challenges involve transportation issues, fear of discrimination, fear of carrying infection home, difficulty staying updated with guidelines, and challenges in convincing patients and caregivers to curtail hospital visits. At the home and hospice level, challenges include limited services provided by NGOs and reduced hospice admissions due to fear of community transmission. By exploring these specific challenges, the study aims to provide a comprehensive understanding of the multifaceted issues physicians face in palliative care, thereby informing strategies to improve support systems and care quality [4].

One of the primary challenges is the emotional and psychological toll on physicians [5]. Dealing with death and dying, managing patient and family expectations, and making critical care decisions can lead to emotional exhaustion and burnout [6]. This aspect of palliative care is critical as it impacts the mental health of physicians and the quality of care they provide [7]. Clinical complexities in palliative care often involve managing severe and multifaceted symptoms [8]. Additionally, ethical dilemmas, such as decisions about life-sustaining treatments, present significant challenges [9, 10].

Effective communication is a cornerstone of palliative care, yet it presents significant challenges for physicians [11]. The difficulties in discussing prognosis, treatment options, and end-of-life preferences with patients and families are compounded by cultural, linguistic, and personal barriers [11].

Broader systemic and institutional challenges also impact the delivery of palliative care [12]. These include limited access to specialized training, resource constraints, and healthcare policies that may not fully support palliative care services [13, 14].

This research aims to explore the challenges faced by Saudi physicians in palliative care from their perspective. Grasping these challenges is essential for creating more effective support systems for healthcare providers, refining palliative care education, and ultimately improving patient and family care. This research seeks to make a meaningful contribution to palliative care and drive positive reforms in the Saudi healthcare system.

## Materials and methods

Study design

This research was conducted as a cross-sectional study, a design ideal for capturing a snapshot of the current challenges faced by physicians in palliative care within the Saudi Arabian healthcare context. The cross-sectional nature of the study allowed for the collection of data at a single point in time, providing insights into the perceptions, experiences, and challenges that physicians encountered in their practice of palliative care.

Study setting

The study was conducted across three prominent healthcare institutions in Riyadh, Saudi Arabia: King Fahad Medical City (KFMC), National Guard Hospital (NGH), and King Faisal Specialist Hospital and Research Center (KFSH&RC). These institutions are renowned for their excellence in healthcare and their comprehensive approach to medical services, making them ideal settings for this research.

KFMC offers specialized palliative care and provides a diverse environment for understanding challenges. The NGH, known for its expert staff and facilities, added insights into palliative care for a wide demographic. KFSH&RC contributed valuable perspectives, combining clinical excellence with research-driven insights.

The study spanned 12 months, commencing in January 2024 and concluding in August 2024, allowing for an in-depth exploration and analysis of palliative care practices across these distinguished medical institutions.

Study population

The study population for this research consisted of physicians working in palliative care across three major healthcare institutions in Riyadh, Saudi Arabia: KFMC, NGH, and KFSH&RC. The total number of physicians working in palliative care across these institutions is approximately 250. Among them, 100 physicians are at KFMC, 80 at NGH, and 70 at KFSH&RC. This group includes physicians from various specialties, such as oncology, internal medicine, and pain management, who are directly involved in providing palliative care, thus offering a diverse range of perspectives and experiences.

Sample size estimation

In determining the appropriate sample size for this study, we employed G*Power statistical software, utilizing specific parameters to ensure the statistical robustness and validity of the results. We set the parameters to include a medium effect size of 0.5, a confidence interval of 95%, an alpha value of 0.05, and a power of 0.95. These parameters were chosen to balance the need for a sample size that was both feasible and sufficient to detect a meaningful effect. Based on these inputs, G*Power calculated a required sample size of 176 participants.

To account for potential issues such as participant dropout or incomplete responses, which are common in survey-based research, we decided to increase the sample size by an additional 15%. This precautionary measure aimed to ensure that the study maintained its statistical power and the ability to draw reliable conclusions, even in the event of participant attrition. Consequently, the final targeted sample size for this study was set to approximately 198 participants.

Inclusion criteria and exclusion criteria

Participants must be licensed physicians currently practicing at KFMC, NGH, or KFSH&RC, actively involved in providing palliative care either as part of a specialized team or through routine integration into their practice. They must have a minimum of one year of experience in palliative care to ensure sufficient practical exposure and must voluntarily agree to participate by providing informed consent. Physicians who do not actively participate in palliative care, have less than a year of experience, or do not consent to participate will be excluded, focusing the study on those with direct and substantial experience in palliative care while adhering to ethical research guidelines.

Data collection

The data collection for this study on palliative care challenges was conducted using an electronic bilingual (Arabic/English) questionnaire, distributed to physicians at KFMC, NGH, and KFSH&RC in Riyadh during the period between May and June. After the development and pilot testing of the questionnaire to ensure its comprehensiveness and clarity, the finalized version was disseminated via social media platforms, primarily WhatsApp, which is widely used by healthcare professionals in Saudi Arabia. This method facilitated easy access and convenience for the participants. An introductory section in the questionnaire provided details about the study, ensured confidentiality, and sought informed consent. The link to the questionnaire, hosted on a secure platform, was shared in relevant social media groups and networks of healthcare professionals, encouraging broad participation over a set period.

Data Collection Tool

The study employed a structured questionnaire based on the tool used in the research by Gupta et al. [15], “A Questionnaire-based Survey on Challenges Faced and Strategies Adopted by Pain and Palliative Care Physicians Working in Oncology Setup.” This questionnaire is particularly apt for exploring the challenges faced by palliative care physicians, making it an ideal instrument for this study.

The questionnaire adapted from Gupta et al. [15] for this study was structured to explore the various challenges faced by physicians in palliative care. Here’s a description of the content of the questionnaire.

Consent to Participate

The first part of the questionnaire included a consent form that outlines the purpose of the study, the voluntary nature of participation, and assurances of confidentiality. Participants must agree to participate before proceeding with the questionnaire.

Personal Details

Age: Physicians were asked to indicate their age range (e.g., 20-40, 40-60, >60 years).

Gender: Options included male and female.

Specialty: Participants selected their medical specialty from a list that includes anesthesia, pain medicine, palliative medicine, critical care, radiation oncology, medical oncology, and surgical oncology among others. There was also an option to specify other specialties not listed.

Years of experience in the specialty: Options will range from 1-3 years, 3-6 years, to more than 6 years.

Place of practice: This section asked the participants to indicate their primary place of practice. They will also be asked to provide the name of the hospital along with the city and state, if applicable.

Challenges at Different Levels

This part of the questionnaire was designed to identify specific challenges faced in palliative care. It will be divided into subsections focusing on different levels of challenges:

Hospital level: Challenges included limited outpatient and inpatient services, access to allied healthcare professionals, ethical dilemmas, lack of pharmacy dispensing wings for pain medications, lack of telemedicine facility, restricted entry to caregivers, and others.

Personal level: Challenges covered issues like transportation to the workplace, fear of discrimination, difficulty in keeping up-to-date with guidelines, and convincing patients and caregivers about limiting hospital visits.

Home and hospice level: This section explored challenges such as limited services provided by NGOs, reduced admissions in hospices, and other relevant issues.

To ensure the suitability of the questionnaire for the Saudi Arabian context, modifications were made based on the original questionnaire. A pilot study was conducted with a group of palliative care physicians to validate the adapted questionnaire. Feedback from this pilot guided further refinements to enhance clarity, relevance, and cultural appropriateness. The input from palliative care experts was also incorporated to ensure the questionnaire’s comprehensiveness and reliability.

The reliability analysis of the 45-item scale, conducted with a sample of 18 participants, indicated a good overall internal consistency, with a Cronbach’s alpha of 0.795. When examining specific domains, the “Challenges Domain” at the hospital level showed a very good internal consistency (Cronbach’s alpha = 0.814 for ten items). The personal-level challenges domain had a Cronbach’s alpha of 0.846 for six items, while the home and hospice level challenges demonstrated excellent internal consistency with a Cronbach’s alpha of 0.923 for three items. In the “Strategies Domain,” system-level strategies yielded a Cronbach’s alpha of 0.734 for eight items, indicating acceptable internal consistency. The staff-level strategies had a good internal consistency with a Cronbach’s alpha of 0.793 for 11 items, and the space-level strategies showed a very good internal consistency (Cronbach’s alpha = 0.820 for three items). Lastly, the strategies at the level of “Stuff” demonstrated excellent internal consistency with a Cronbach’s alpha of 0.884 for four items. These results suggest that the scale and its subdomains have reliable internal consistency, making them suitable for assessing the relevant challenges and strategies in palliative care.

Ethical considerations

In addressing the ethical considerations for this study, we strictly adhered to the highest standards throughout the research process. Ethical approval was sought from the institutional review board (IRB) of KFMC (IRB tog number: 24-064), as this was the only institution among the three settings that required such approval. This process ensured that the study’s design, including data collection methods and participant engagement, aligned with the required ethical guidelines.

Confidentiality and anonymity of participants were a top priority. Personal identifiers were not collected. If they were necessary for the study, they were securely stored and accessed solely by the research team. Informed consent was a crucial part of the participant engagement process. All participants were provided with clear information about the study’s purpose, their voluntary involvement, and their right to withdraw at any time without repercussions.

The study also guaranteed that the data collected was used exclusively for the research objectives. Any dissemination of the findings, whether through reports or publications, was conducted in a manner that safeguarded individual identities and maintained the confidentiality of the participants. This commitment to ethical research practice was fundamental to the integrity and credibility of the study.

Data management and statistical analysis

For the data management and analysis in this study, the collected data were systematically organized and analyzed using the Statistical Package for the Social Sciences (SPSS). This software was adept at handling large datasets and provided a range of statistical tools suitable for comprehensive analysis. The primary statistical tests employed included descriptive statistics for demographic data and participants’ responses to the scale items. The significance level was set at p < 0.05 to determine statistical significance.

## Results

Baseline socio-demographic characteristics of the enrolled physicians

The age distribution of the enrolled physicians showed that a majority were between 20 and 40 years old, comprising 48.48% (n = 96) of the participants. Physicians aged 40-60 years accounted for 47.47% (n = 94) of the sample, while those over 60 years were the least represented, with 4.04% (n = 8) (Table 1).

**Table 1:** Baseline socio-demographic characteristics of the enrolled physicians

Variable	Frequency	Percentage
Age (Years)		
20-40	96	48.48
40-60	94	47.47
>60	8	4.04
Gender		
Male	128	64.65
Female	70	35.35
Specialty		
Anesthesia	28	14.14
Pain medicine	20	10.1
Palliative medicine	22	11.11
Critical care	31	15.66
Radiation oncology	33	16.67
Medical oncology	21	10.61
Surgical oncology	25	12.63
Other	18	9.09
Years of experience		
1-3 years	65	32.83
3-6 years	80	40.4
>6 years	53	26.77
Place of practice		
King Fahad Medical City (KFMC)	73	36.87
National Guard Hospital (NGH)	81	40.91
King Faisal Specialist Hospital and Research Center (KFSH&RC)	44	22.22

In terms of gender, the sample had a higher percentage of male physicians, with 64.65% (n = 128) identifying as male, compared to 35.35% (n = 70) identifying as female (Table 1). The specialties of the enrolled physicians were diverse. Anesthesia specialists made up 14.14% (n = 28) of the participants. Pain medicine specialists accounted for 10.1% (n = 20), while those in palliative medicine represented 11.11% (n = 22). Physicians specializing in critical care comprised 15.66% (n = 31) of the sample, and those in radiation oncology were 16.67% (n = 33). Medical oncology specialists made up 10.61% (n = 21), surgical oncology specialists represented 12.63% (n = 25), and those in other specialties accounted for 9.09% (n = 18) (Table 1).

Regarding years of experience, 32.83% (n = 65) of the physicians had 1-3 years of experience, 40.4% (n = 80) had 3-6 years, and 26.77% (n = 53) had more than 6 years of experience (Table 1). The place of practice was also varied among the physicians. Those practicing at KFMC comprised 36.87% (n = 73) of the sample, while 40.91% (n = 81) were from the NGH, and 22.22% (n = 44) practiced at the KFSH&RC (Table 1).

Challenges faced at the “hospital level”

As shown in Table 2, various challenges were identified at the hospital level. Limited outpatient and inpatient services were reported by 54.55% (n = 108) of the physicians. A higher percentage, 60.61% (n = 120), indicated limited access to allied healthcare professionals as a significant challenge. Ethical dilemmas due to triaging were noted by 63.13% (n = 125) of the respondents.

**Table 2:** Challenges faced by the enrolled physicians at the hospital level (n=198)

Challenge	Frequency	Percentage
Limited outpatient and inpatient services	108	54.55
Limited access to allied healthcare professionals	120	60.61
Ethical dilemma due to triaging	125	63.13
Lack of pharmacy dispensing wings	107	54.04
Lack of telemedicine facility	113	57.07
Restricted entry to caregivers	127	64.14
Pain and palliative care research affected by the pandemic	119	60.1
An inadequate place to screen suspected COVID-19 patients	122	61.62
Attending COVID-19 positive case for symptom management	118	59.6

The lack of pharmacy dispensing wings was cited by 54.04% (n = 107) of the physicians, while 57.07% (n = 113) reported the lack of a telemedicine facility as a challenge. Restricted entry to caregivers was the most frequently reported issue, with 64.14% (n = 127) of physicians acknowledging it. The impact of the pandemic on pain and palliative care research was highlighted by 60.1% (n = 119) of the respondents.

Additionally, 61.62% (n = 122) of the physicians identified the inadequate place to screen suspected COVID-19 patients as a challenge. Finally, attending to COVID-19 positive cases for symptom management was reported by 59.6% (n = 118) of the physicians.

Challenges faced by the enrolled physicians at the personal level

As shown in Table 3, the enrolled physicians faced several challenges at the personal level. To and from transportation to the workplace was reported by 54.55% (n = 108) of the physicians. Fear of discrimination by society was noted by 60.61% (n = 120) of the respondents, while the fear of carrying infection back home was a concern for 63.13% (n = 125) of the physicians.

Keeping updated with the latest guidelines was identified as a challenge by 54.04% (n = 107) of the physicians. Additionally, 57.07% (n = 113) of the respondents reported difficulty in convincing patients and caregivers about curtailing their visits to the hospital.

**Table 3:** Challenges faced by the enrolled physicians at the personal level (n=198)

Challenge	Frequency	Percentage
To and from transportation to the workplace	108	54.55
Fear of discrimination by society	120	60.61
Fear of carrying infection back home	125	63.13
Keeping yourself updated with all the latest guidelines	107	54.04
Difficulty in convincing patients and caregivers	113	57.07

Challenges faced by the enrolled physicians at the home and hospice level

As shown in Table 4, the enrolled physicians faced specific challenges at the home and hospice level. Limited services provided by NGOs during the pandemic were reported by 54.55% (n = 108) of the physicians. Additionally, 60.61% (n = 120) of the respondents noted reduced admissions by hospices due to the fear of community transmission.

**Table 4:** Challenges faced by the enrolled physicians at the home and hospice level (n=198) NGOs: non-governmental organizations

Challenge	Frequency	Percentage
Limited services provided by NGOs during this pandemic	108	54.55
Reduced admissions by the hospices due to fear of community transmission	120	60.61

System-level strategies adopted by the enrolled physicians

As shown in Table 5, various system-level strategies were adopted by the enrolled physicians to improve pain and palliative care. Creating a triage system was implemented by 54.55% (n = 108) of the physicians. The use of telemedicine was reported by 60.61% (n = 120) of the respondents, while advanced care planning was adopted by 63.13% (n = 125) of the physicians.

A system for the transfer of patients requiring dedicated palliative care was created by 54.04% (n = 107) of the physicians. Utilizing the services of NGOs was a strategy used by 57.07% (n = 113) of the respondents. Paid leave for healthcare workers was provided by 64.14% (n = 127) of the physicians. Finally, providing necessary personal protective equipment (PPE) equipment was a strategy adopted by 60.1% (n = 119) of the respondents.

**Table 5:** System-level strategies adopted by the enrolled physicians PPE: personal protective equipment; NGOs: non-governmental organizations

Strategy	Frequency	Percentage
Created a triage system	108	54.55
Use of telemedicine	120	60.61
Advanced care planning	125	63.13
Creating a system for the transfer of patients	107	54.04
Utilizing the services of NGOs	113	57.07
Paid leave for healthcare workers	127	64.14
Providing necessary PPE equipment	119	60.1

Staff-level strategies adopted by the enrolled physicians

As shown in Table 6, several staff-level strategies were adopted by the enrolled physicians to enhance the delivery of pain and palliative care. The formation of a task force was reported by 61.62% (n = 122) of the physicians. Educational sessions for the task force were conducted by 59.6% (n = 118) of the respondents, while training sessions for patients and caregivers were provided by 62.12% (n = 123) of the physicians.

**Table 6:** Staff-level strategies adopted by the enrolled physicians

Strategy	Frequency	Percentage
Formation of the task force	122	61.62
Educational sessions for the task force	118	59.6
Training sessions for patients and caregivers	123	62.12
Development of standardized protocols	124	62.63
Use of a screening tool for triaging	118	59.6
Involving allied healthcare workers	121	61.11
Use of screening stations for new patients	110	55.56
Use of screening stations for influenza-like illness	124	62.63
Use of teleconsultation	117	59.09
Empowering family caregivers	116	58.59

The development of standardized protocols was undertaken by 62.63% (n = 124) of the physicians. The use of a screening tool for triaging was implemented by 59.6% (n = 118) of the respondents. Involving allied healthcare workers in providing psychosocial and spiritual support was a strategy used by 61.11% (n = 121) of the physicians.

The use of screening stations for new patients was reported by 55.56% (n = 110) of the physicians, while screening stations for influenza-like illness were used by 62.63% (n = 124) of the respondents. Tele-consultation for patients under follow-up was utilized by 59.09% (n = 117) of the physicians. Finally, empowering family caregivers to play an active role in providing care was a strategy adopted by 58.59% (n = 116) of the respondents.

Space-level strategies adopted by the enrolled physicians

As shown in Table 7, the enrolled physicians adopted several strategies at the space level to improve pain and palliative care. Identification of areas in the facility to provide pain and palliative care was implemented by 58.59% (n = 116) of the physicians. Additionally, maximum utilization of available space, following the norms of social distancing, was reported by 57.58% (n = 114) of the respondents.

**Table 7:** Space-level strategies adopted by the enrolled physicians

Strategy	Frequency	Percentage
Identification of areas in the facility	116	58.59
Maximum utilization of available space	114	57.58

Stuff-level strategies adopted by the enrolled physicians

As shown in Table 8, various strategies related to “stuff” were adopted by the enrolled physicians to ensure the availability of essential resources for pain and palliative care. Stockpiling medications for common symptoms was reported by 59.6% (n = 118) of the physicians. Additionally, stockpiling necessary equipment was a strategy used by 57.07% (n = 113) of the respondents. The provision of medication and equipment kits for home care was implemented by 63.13% (n = 125) of the physicians.

**Table 8:** Stuff-level strategies adopted by the enrolled physicians

Strategy	Frequency	Percentage
Stockpile of medications	118	59.6
Stockpile of necessary equipment	113	57.07
Medication and equipment kits for home care	125	63.13

## Discussion

This study aimed to explore and identify the multifaceted challenges faced by physicians in palliative care within three major healthcare institutions in Riyadh, Saudi Arabia. By examining these challenges at the hospital, personal, and home and hospice levels, the research seeks to inform the development of targeted support systems and improvements in palliative care services. The findings of this study highlight several significant challenges faced by physicians in palliative care within the Saudi Arabian healthcare context. The data revealed that limited outpatient and inpatient services, restricted access to allied healthcare professionals, and ethical dilemmas due to triaging were major issues. These challenges resonate with previous studies that have identified similar barriers in palliative care settings globally [1]. In particular, the lack of adequate services and access to multidisciplinary care teams can impede the delivery of comprehensive palliative care.

Ethical dilemmas, particularly those arising from triaging decisions, were prominently reported by the participants. This challenge is consistent with findings from other regions, where ethical complexities often arise in palliative care due to the need to balance resource allocation and patient needs [8]. The COVID-19 pandemic has exacerbated these ethical challenges, highlighting the need for robust ethical guidelines and support systems for healthcare providers [15].

The study also identified a significant concern regarding the lack of telemedicine facilities, which has become an essential component of healthcare delivery during the COVID-19 pandemic. Telemedicine can bridge the gap in healthcare access, especially in palliative care, where frequent consultations are necessary. The adoption of telemedicine in palliative care has been shown to improve patient outcomes and satisfaction [13]. However, the lack of such facilities in Saudi Arabia underscores a critical area for improvement.

Restricted entry to caregivers was another significant challenge reported by the physicians. This restriction, while necessary for infection control, can negatively impact the psychosocial support provided to patients and their families, which is a cornerstone of palliative care [4]. The importance of family involvement in palliative care cannot be overstated, as it provides emotional support and improves the overall well-being of patients [11].

The impact of the pandemic on pain and palliative care research was also notable, with many physicians reporting disruptions. This finding aligns with global reports on the challenges faced by palliative care researchers during the pandemic, which include difficulties in conducting clinical trials and accessing research funding [7]. Ensuring the continuity of palliative care research is crucial for the development of evidence-based practices and improving patient care.

Personal-level challenges, such as fear of discrimination and infection, were prevalent among the physicians. These concerns are not unique to Saudi Arabia and have been reported in various settings, particularly during the COVID-19 pandemic [10]. Addressing these fears through adequate protective measures and support systems is essential to maintain the mental health and well-being of healthcare providers.

The study highlighted the strategies adopted at different levels to mitigate these challenges. System-level strategies, such as the creation of triage systems and the use of telemedicine, were commonly implemented. These strategies are supported by literature as effective means to streamline palliative care delivery and enhance patient care [5]. However, the implementation of these strategies requires adequate resources and training, which were identified as areas needing improvement.

Staff-level strategies, including the formation of task forces and conducting educational sessions, were also reported. These initiatives are crucial for building a competent workforce capable of addressing the complexities of palliative care [6]. Training programs and continuous education can empower healthcare providers with the necessary skills and knowledge to deliver high-quality care [14].

Finally, the identification of areas within healthcare facilities for palliative care and the maximum utilization of available space were important space-level strategies. These measures are essential for ensuring that palliative care services are accessible and well-integrated within the healthcare system [3]. The study’s findings underscore the need for a multifaceted approach to address the challenges in palliative care, involving systemic, staff, and infrastructural improvements.

To sum up, this study provides valuable insights into the challenges and strategies in palliative care within the Saudi Arabian context. The findings highlight the need for comprehensive approaches to enhance palliative care services, including the adoption of telemedicine, ethical support for healthcare providers, and continuous education and training. These measures are essential for improving the quality of palliative care and ensuring that patients receive the best possible support in managing their conditions.

This study has several limitations that should be considered when interpreting the findings. First, the cross-sectional design captures only a snapshot of the challenges and strategies at a single point in time, which may not reflect ongoing changes or developments in palliative care practices. Second, the reliance on self-reported data from physicians may introduce response bias, as participants might overestimate or underestimate certain challenges or strategies. Additionally, the study was conducted in three major healthcare institutions in Riyadh, which may limit the generalizability of the results to other regions or smaller healthcare settings in Saudi Arabia. Finally, the use of electronic questionnaires distributed via social media platforms, while convenient, may have excluded physicians who are less engaged with these platforms, potentially affecting the representativeness of the sample.

## Conclusions

This study provides valuable insights into the challenges faced by physicians in palliative care within the Saudi Arabian healthcare context and the strategies they adopt to mitigate these issues. The findings highlight significant barriers, including limited services, ethical dilemmas, lack of telemedicine facilities, and personal fears related to the COVID-19 pandemic. The study also underscores the importance of implementing system-level, staff-level, and infrastructural strategies to enhance palliative care delivery. Addressing these challenges requires a comprehensive approach that includes ethical support for healthcare providers, the integration of telemedicine, continuous education and training, and improved access to multidisciplinary care teams. These measures are essential for improving the quality of palliative care and ensuring that patients receive the holistic support they need.

## Appendices

Exploring the challenges in palliative care as perceived by the Saudi Physicians

Personal Details

1. Age (Years)*

Mark only one oval.

◯ 20-40

◯ 40-60

◯ >60

2. Gender*

Mark only one oval.

◯ Male

◯ Female

3. Specialty (Multiple options can be marked depending upon the practice)*

Tick all that apply.

□ Anesthesia

□ Pain medicine

□ Palliative medicine

□ Critical care

□ Radiation oncology

□ Medical oncology

□ Surgical oncology

Other: □ ___________________

4. Years of experience in the specialty*

Mark only one oval.

◯ 1-3 Years

◯ 3-6 Years

◯ >6 Years

5. Place of practice*

Mark only one oval.

◯ King Fahad Medical City (KFMC)

◯ National Guard Hospital (NGH)

◯ King Faisal Specialist Hospital and Research Center (KFSH&RC)

Challenges at Different Levels

You can choose more than one option (Tick all that apply)

6. Challenges face at “hospital level”*

Tick all that apply.

□ Limited outpatient and inpatient services

□ Limited access to allied healthcare professionals like physiotherapist and wound care specialist

□ Ethical dilemma because of triaging of patients leaving many patients with mild and moderate symptoms unattended

□ Lack of pharmacy dispensing wings in various hospitals to deliver pain medications like opioid to patients

□ Lack of telemedicine facility

□ Restricted entry to the caregivers impedes the ability to provide needed psychosocial support to family members which is of utmost importance in cancer patients

□ Pain and palliative care research in cancer patients getting affected due to the pandemic.

□ Inadequate place to screen the suspected corona patients and inadequate testing.

□ There is a possibility of attending a COVID-19-positive case for symptom management in the palliative care services provided

Other: □ ___________________

7. Challenges at “personal level”*

Tick all that apply.

□ To and fro transportation to the working place

□ Fear of discrimination by society

□ Fear of carrying infection back home

□ Keeping yourself updated with all the latest guidelines related to COVID-19 and cancer care becomes difficult

□ Difficulty in convincing patients and caregivers aware about curtailing their visits to hospital

Other: □ ___________________

8. Challenges at the “level of home and hospice” based pain and palliative services* (Tick all that apply)

□ Limited services provided by NGOs during this pandemic because of the lack of their support staff, inability to go to patients' homes and limited availability of PPE with them

□ Reduced admissions by the hospices due to fear of community transmission.

Other: □ ___________________

Strategies Adopted at Different Levels

You can choose more than one option (Tick all that apply)

9. “System-level” strategies* (Tick all that apply)

□ Created a triage system to identify patients in need of specialist pain and palliative care

□ Use of telemedicine for direct consultation support for the patients and their family members

□ Advanced care planning is done for all the patients admitted to the hospital setting

□ Creating a system for transfer of the patients who require dedicated palliative care and hospice services.

□ Utilizing services of non-governmental organizations for helping patients in need of pain and palliative care services.

□ Paid leave for some proportion of healthcare workers so that in case of inadvertent need of quarantine of those affected there is always a pool of workforce left to provide health services.

□ Providing necessary PPE equipment like N95 masks and hydroxychloroquine tablets to the healthcare workers in the department

Other: □ ___________________

10. “Staff-level” strategies* (Tick all that apply)

□ Formation of the task force for providing pain and palliative care

□ Conduct educational sessions for the task force members during the COVID pandemic

□ Conduct training sessions for the patient and their caregivers explaining to them about the pandemic and the precautions they need to follow

□ Development of standardized protocols for symptom management

□ Use of any screening tool for further triaging the palliative care needs of patients into priority A, B, and C

□ Involving the specialized allied healthcare workers in providing psychosocial and spiritual support

□ Use of screening stations to segregate new patients and those who are on follow-up

□ Use of screening stations to identify patients with influenza-like illness or severe acute respiratory illness so that they can be diverted to dedicated COVID-19 care facility

□ Use of teleconsultation for every patient under follow-up

□ Empowering family caregivers to play an active role in providing care

Other: □ ___________________

11. “Space-level” strategies* (Tick all that apply)

□ Identification of areas in the health care facility to provide pain and palliative care

□ Maximum utilization of the available space following the norms of social distancing amongst the patients and health care workers at the same point of time

Other: □ ___________________

12. Strategies at the level of “Stuff ”* (Tick all that apply)

□ Stockpile of medications for common symptoms like - pain, dyspnea, nausea, vomiting, delirium, and secretions

□ Stockpile of necessary equipment to deliver medications like subcutaneous butterflies and drug delivery pumps

□ Medication and equipment kits for home care and long-term care facility

Other: □ ___________________
